# Alpha Ketoglutarate Downregulates the Neutral Endopeptidase and Enhances the Growth Inhibitory Activity of Thiorphan in Highly Aggressive Osteosarcoma Cells

**DOI:** 10.3390/molecules28010097

**Published:** 2022-12-22

**Authors:** Magdalena Mizerska-Kowalska, Adrianna Sławińska-Brych, Emilia Niedziela, Viktor Brodovskiy, Barbara Zdzisińska

**Affiliations:** 1 Department of Virology and Immunology, Faculty of Biology and Biotechnology, Maria Curie-Skłodowska University, Akademicka 19 Street, 20-033 Lublin, Poland; 2 Department of Cell Biology, Faculty of Biology and Biotechnology, Maria Curie-Skłodowska University, Akademicka 19 Street, 20-033 Lublin, Poland

**Keywords:** neutral endopeptidase, α-ketoglutarate, epigenetic regulation, natural anticancer products, enhancer of zeste homolog 2 (EZH2) methyltransferase, combination therapies

## Abstract

Since natural substances are widely explored as epigenetic modulators of gene expression and epigenetic abnormalities are important causes of cancerogenesis, factors with pro-tumor activities subjected to epigenetic control, e.g., neutral endopeptidase (NEP, neprilysin), are promising anticancer targets for potential therapies acting via epigenetic regulation of gene expression. Alpha-ketoglutarate (AKG) is a naturally occurring co-substrate for enzymes involved in histone and DNA demethylation with suggested anti-cancer activity. Hence, we investigated a potential effect of AKG on the NEP expression in cells derived from various cancers (cervical, colon, osteosarcoma) and normal epithelial cells and osteoblasts. Moreover, the overall methylation status of histone H3 was explored to establish the molecular target of AKG activity. Additionally, it was investigated whether AKG in combination with thiorphan (NEP specific inhibitor) exhibited enhanced anticancer activity. The results revealed that AKG downregulated the expression of NEP at the protein level only in highly aggressive osteosarcoma HOS cells (flow cytometry and fluorometric assays), and this protease was found to be involved in AKG-induced growth inhibition in osteosarcoma cells (siRNA NEP silencing, BrdU assay, flow cytometry). Unexpectedly, AKG-induced hypermethylation of H3K27 in HOS cells, which was partially dependent on EZH2 activity. However, this effect was not implicated in the AKG-induced NEP downregulation (flow cytometry). Finally, the combined treatment with AKG and thiorphan was shown to significantly enhance the growth inhibitory potential of each one towards HOS cells (BrdU assay). These preliminary studies have shown for the first time that the downregulation of NEP expression is a promising target in therapies of NEP-implicating HOS cells. Moreover, this therapeutic goal can be achieved via AKG-induced downregulation of NEP and synergistic activity of AKG with thiorphan, i.e., a NEP specific inhibitor. Furthermore, this study has reported for the first time that exogenous AKG can influence the activity of histone methyltransferase, EZH2. However, this issue needs further investigation to elucidate the mechanisms of this phenomenon.

## 1. Introduction

Despite the considerable progress in the prevention, diagnostics, and treatment of cancers, high rates of recurrence and morbidity are still observed [1]. Hence, investigations of new cancer preventive or curing substances are still an important field in biomedicine. Along with surgery and radiotherapy, chemotherapy is still the main method of cancer treatment. Drugs based on natural products together with semi-synthetic and synthetic agents constitute diverse groups of medicines used in cancer treatment. One of the groups includes mainly unaltered or chemically modified natural products and drugs based on naturally occurring pharmacophores [2]. Alpha-ketoglutarate (AKG) is a naturally occurring molecule synthesized as an endogenous intermediary metabolite in the Krebs cycle by animal and bacterial cells. This molecule serves diverse intra- and extracellular functions and can therefore be viewed as a promising natural supplement in prevention, chemosensitization, and treatment of age-related disorders and cancers [3,4]. Its pleiotropic activities results, among other things, from the involvement of AKGas a co-substrate for 2-oxoglutarate-dependent dioxygenases (2-OGDDs) in the epigenetic regulation of gene expression.

Disturbances in the genetic and epigenetic regulation of gene expression are the main causative events that lead, e.g., to the disparate expression of molecules in neoplastic cells in comparison with their normal counterparts. For example, it has been shown that the level of neutral endopeptidase (NEP, neprilysin, CD10, CALLA, EC 3.4.21.11) is elevated or decreased in cancer cells, in comparison with normal ones, or varies between the different tumor stages [5,6,7,8]. This zinc-dependent proteolytic enzyme has different forms, namely it can be cell-associated or soluble in body fluids, e.g., blood and cerebrospinal fluid. The cell-associated form of NEP is present in a wide range of cells, e.g., epithelial cells and fibroblasts of different organs, as well as nerve and immune cells. The widespread expression results in engagement of neprilysin in diverse physiological (e.g., lymphocyte B maturation) and pathological (e.g., neurodegeneration or tumorigenesis) processes [9,10,11,12,13]. Although it acts via proteolytic regulation of the level of bioactive peptides and proteins, the NEP enzyme also influences signaling pathways by direct interaction with their components, e.g., PTEN, Lyn, and ERM proteins [12,13,14,15]. It has been evidenced that the biological functions of NEP can be regulated at both the expression and enzymatic levels, which can be exploited as therapeutic strategies, for instance, in cancer treatment [10,11,12,13,16]. The expression is subjected to epigenetic regulation. It is mediated via changes in the methylation of CpG islands in the promoter region of NEP and changes in the methylation/acetylation of histone H3 [13,17,18]. In turn, there are synthetic (e.g., sacubitril, thiorphan, and α-aminophosphonous acid derivatives) and natural (e.g., sialorphin) inhibitors of NEP that are used as medicines or have exhibited therapeutic potential in in vitro and in vivo studies [5,19,20,21,22,23].

There have been many attempts to exploit NEP as a therapeutic target, e.g., in cancers. However, since the role of NEP in the development of any disorders seems to be dependent on its expression level, the effect of treatment, i.e., up- or downregulation of NEP should be considered [12,13,17]. There are studies indicating a high level of NEP expression in cancer cells, e.g., colon cancer and osteosarcoma. Hence, in these cases, NEP inhibitors or molecules downregulating NEP expression seem to exert an anti-cancer effect [5,8,22,23,24]. Recently, it has been shown that specific inhibitors of NEP, i.e., synthetic thiorphan, sialorphin based on natural products, and newly discovered derivatives of α-aminophosphonous acid, have direct growth inhibitory activity against cancer cells, e.g., colon cancer, glioma cells, and osteosarcoma [22,23,25]. However, to the best of our knowledge, there are no other studies on naturally occurring molecules that are able to downregulate NEP expression as an anticancer therapeutic target [12,13,17]. This prompted us to investigate whether AKG has an influence on the level of NEP in cells derived from different types of tumors. Moreover, taking into account the epigenetic regulation of NEP expression and the involvement of AKG in this type of processes, the overall histone H3 methylation status in cancer cells upon AKG treatment was explored to establish the molecular target of AKG activity. Additionally, the effect of combined treatment of AKG and the NEP inhibitor, i.e., thiorphan, towards cancer cells was investigated, as the latter compound has been proven to have promising but moderate anticancer potential [22,23].

## 2. Results

### 2.1. AKG Downregulated the Protein Level and Activity of NEP in Highly Aggressive Osteosarcoma Cells

**Figure 1:**
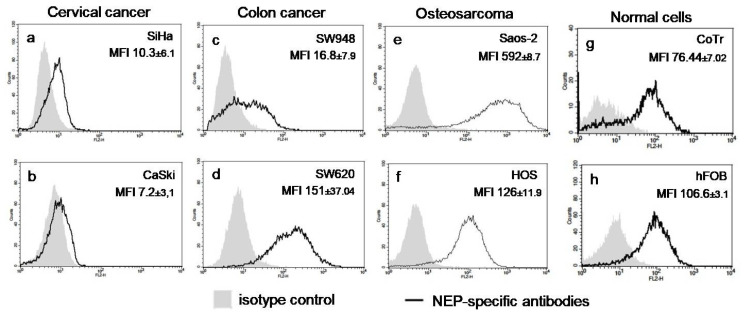
Expression of NEP at the protein level in primary (SiHa) (**a**) and metastatic (CaSki) (**b**) cervical cancer cells, primary (SW948) (**c**) and metastatic (SW620) (**d**) colon cancer cells, primary moderately (Saos-2) (**e**) and highly (HOS) (**f**) aggressive osteosarcoma cells, normal epithelial cells of the intestine (CoTr) (**g**), and normal osteoblasts (hFOB) (**h**). The level of NEP was quantified by means of flow cytometry after cell staining with the PE-conjugated mouse IgG1 isotype control and with PE-conjugated mouse anti-human NEP mAb IgG1. The level of NEP was expressed as Mean Fluorescence Intensity (MFI) ± SD of three independent experiments. The histograms shown are representative.

It is known that the expression of NEP is regulated epigenetically [17,18,26]. In turn, as an intracellular factor, AKG is involved in the regulation of this type of process [27,28,29]. Hence, the protein level and activity of NEP was examined in six cancer cell lines, i.e., SiHa and CaSki (primary and metastatic cervical cancer, respectively); SW948 and SW620 (primary and metastatic colon cancer, respectively); HOS and Saos-2 (primary highly and moderately aggressive osteosarcoma) [30], and in normal epithelial cells of the intestine CCD 841 CoTr and osteoblasts hFOB after AKG treatment to determine the potential influence of AKG on NEP expression. The intrinsic level of NEP in the analyzed cell lines has been reported previously [5,23] and indicated in this study. In general, the flow cytometry analysis showed that the cervical cancer CaSki cells did not express NEP (Figure 1b), whereas the other cancer and normal cells expressed neprilysin; however, they exhibited different patterns in relation to the NEP protein level. Namely, the cervical cancer cells SiHa (MFI = 10.3 ± 6.1) (Figure 1a) and the colon cancer SW948 cells (MFI = 16.8 ± 7.9) (Figure 1c) had the lowest levels of NEP expression in comparison with the intermediate levels of NEP in the colon cancer cells SW620 (MFI = 151 ± 7.04) (Figure 1d) and the osteosarcoma HOS cells (MFI = 126 ± 11.9) (Figure 1f), and finally in comparison with the highest level of NEP in the osteosarcoma cells Saos-2, (MFI = 592 ± 8.7) (Figure 1e). On the other hand, both the normal counterparts of the colon cancer and osteosarcoma cells exhibited comparably moderate levels of NEP expression (CoTr, MFI = 76.44 ± 7.02 and hFOB MFI = 106.6 ± 6±3.1, respectively) (Figure 1g and h).

**Figure 2:**
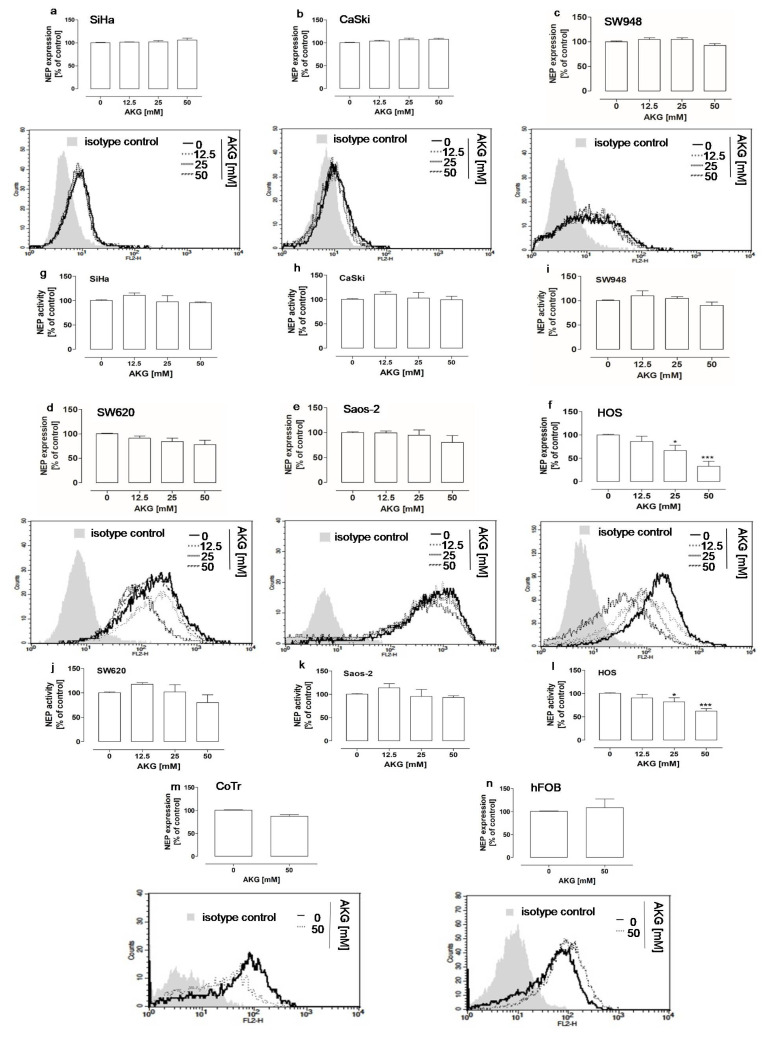
AKG downregulated the protein level and activity of NEP in highly aggressive osteosarcoma cells. The expression at the protein level (**a**–**f**,**m**,**n**) and the activity level (**g**–**l**) of NEP in cervical cancer (**a**,**g**,**b**,**h**), colon cancer (**c**,**i**,**d**,**j**), and osteosarcoma (**e**,**k**,**f**,**l**) cells, and the level of NEP in normal epithelial (CoTr) (**m**) and normal osteoblast (hFob) cells (**n**) after 72-h treatment with the indicated concentrations of AKG. The NEP protein level was determined by means of flow cytometry after staining with PE-conjugated mouse anti-human NEP mAb IgG1. The control cells (0 mM) were also stained with the PE-conjugated mouse IgG1 isotype control. The histograms are representative and correspond with the results presented in the graphs. The activity of NEP was measured by means of a fluorometric assay. The results were expressed as a percent of the control (0 mM) ± SD of three independent experiments. Statistically significant differences: * *p* < 0.05, *** *p* < 0.001 in comparison with the control (unpaired T-test).

Next, the cancer cells and the normal cells were treated with non-cytotoxic concentrations, i.e., 12.5 mM, 25 mM, and/or 50 mM of AKG, respectively, to examine the influence of AKG on NEP expression. These concentrations were chosen according to Żurek 2019 and Kaławaj 2020 (for hFOB, HOS, and Saos-2 cell lines) and were established in the present study (for SiHa, CaSki, SW948, SW620, and CoTr cell lines; Supplementary Figure S1). The flow cytometry analysis revealed that AKG significantly influenced the NEP protein level only in the HOS cells (Figure 2f). The neprilysin level decreased by approx. 33% and 68% in the osteosarcoma cells after the treatment with 25 mM and 50 mM AKG, respectively, in comparison with the control cells. The results of the study on the NEP activity in the AKG-treated cells were consistent with the findings reported in the aforementioned studies. Namely, a statistically significant approx. 18% and 38% decrease in the NEP activity was observed only in the HOS cells after the treatment with 25 mM and 50 mM AKG, respectively, in comparison with the control cells (Figure 2l). In turn, the NEP protein level and activity were unaffected, i.e., neither down- nor upregulated, by the AKG treatment in the other cancer cells, i.e., SiHa (Figure 2a,g), CaSki (Figure 2b,h), SW948 (Figure 2a,e), SW620 (Figure 2b,f), and Saos-2 (Figure 2c,g). Similarly, the level of NEP was not changed after the AKG treatment in the normal epithelial cells (Figure 2i) and osteoblasts (Figure 2j).

### 2.2. The AKG-Mediated EZH2-Dependent Hypermethylation of H3K27 Was Not Implicated in the AKG-Induced NEP Downregulation in the HOS Cells

**Figure 3:**
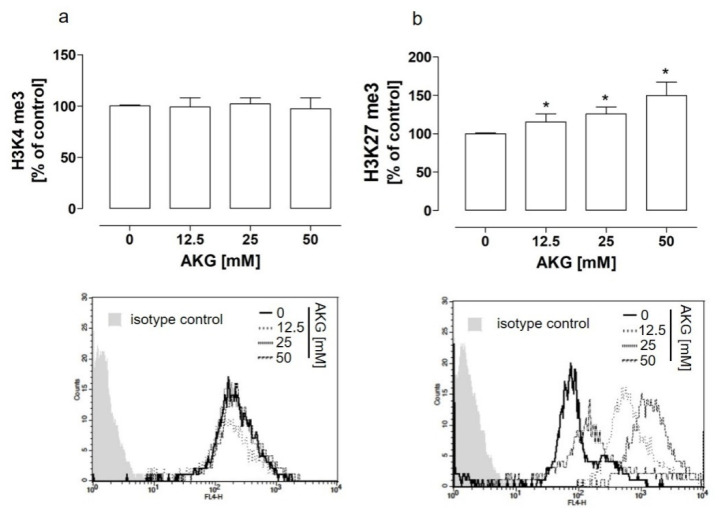
AKG increased the hypermethylation level of lysine 27 in histone H3 via EZH2 methyltransferase in osteosarcoma cells. The HOS cells were treated with the indicated concentrations of AKG for 72 h. The me3 modifications of histone H3 at lysine 4 (**a**) and 27 (**b**) were detected by means of specific Alexa Fluor 447-conjugated antibodies and quantified by flow cytometry. The control cells (0 mM) were also stained with the Alexa Fluor 447-conjugated isotype control. The histograms are representative and correspond with the results presented in the graphs. The results were expressed as a percent of the control (0 mM) ± SD of three independent experiments. Statistically significant differences: * *p* < 0.05 in comparison with the control cells (unpaired T-test).

Taking into account the aforementioned results and the role of AKG as a co-substrate of histone demethylases, to elucidate the mechanism of the AKG-induced NEP downregulation in the HOS cells, further studies were aimed at the examination ofthe methylation status of histone H3 at two different positions, i.e., lysine 4 and 27, whichresulted in opposite effects, i.e., activation and inactivation of gene transcription, respectively. The results indicated that the treatment of the HOS cells with AKG did not change the methylation level at the H3K4 position (Figure 3a), in contrast to the H3K27 position (Figure 3b). Surprisingly, a significant increase in the level of trimethylation of lysine 27 was detected (Figure 3b).

**Figure 4:**
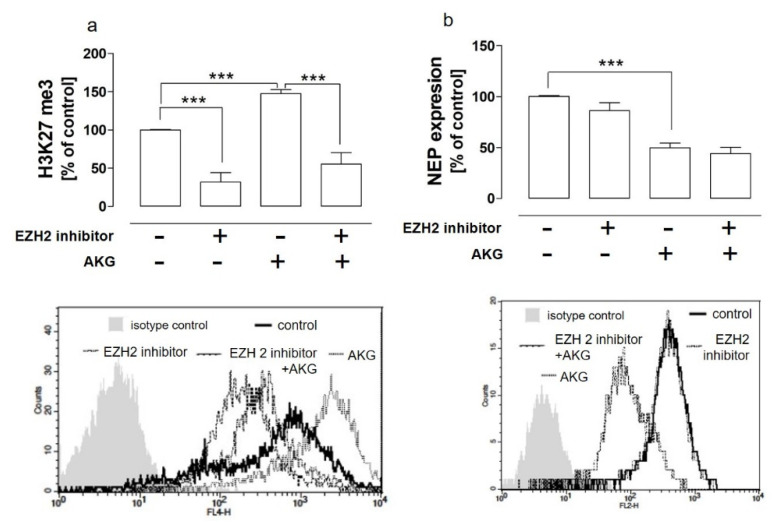
The enhancer of zeste homolog 2 (EZH2) methyltransferase was partially implicated in the AKG-induced hypermethylation of lysine 27 but not in the downregulation of NEP in the osteosarcoma cells. The HOS cells were treated with AKG (50 mM) and/or the EZH2 inhibitor EPZ005687 (10 µM) for 72 h. The me3 modification of histone H3 at lysine 27 (**a**) and NEP expression (**b**) were detected as described in Figure 2 and Figure 3, respectively. The histograms are representative and correspond with the results presented in the graphs. The results were expressed as a percent of the control (0 mM) ± SD of three independent experiments. Statistically significant differences: *** *p* < 0.001, in comparison with the control cells or with the AKG-treated cells (one-way ANOVA followed by Tukey’s post-hoc test).

This unexpected result prompted us to carry out further studies to elucidate whether the enhancer of zeste homolog 2 (EZH2) methyltransferase implicated in lysine 27 hypermethylation was involved in this AKG-induced effect in the HOS cells and whether this mechanism was involved in the AKG-mediated downregulation of NEP. The results presented in Figure 4a indicated that the pretreatment of the HOS cells with the EZH2 specific inhibitor (10 µm) partially abolished the AKG-induced hypermethylation of lysine 27; however, no effect of EZH2 inhibition was observed in the HOS cells in the case of the AKG-induced NEP downregulation (Figure 4b).

### 2.3. The Downregulation of NEP Inhibited Proliferation, Induced Apoptosis, and Was Involved in AKG-Induced Growth Inhibitionin the Osteosarcoma Cells

**Figure 5:**
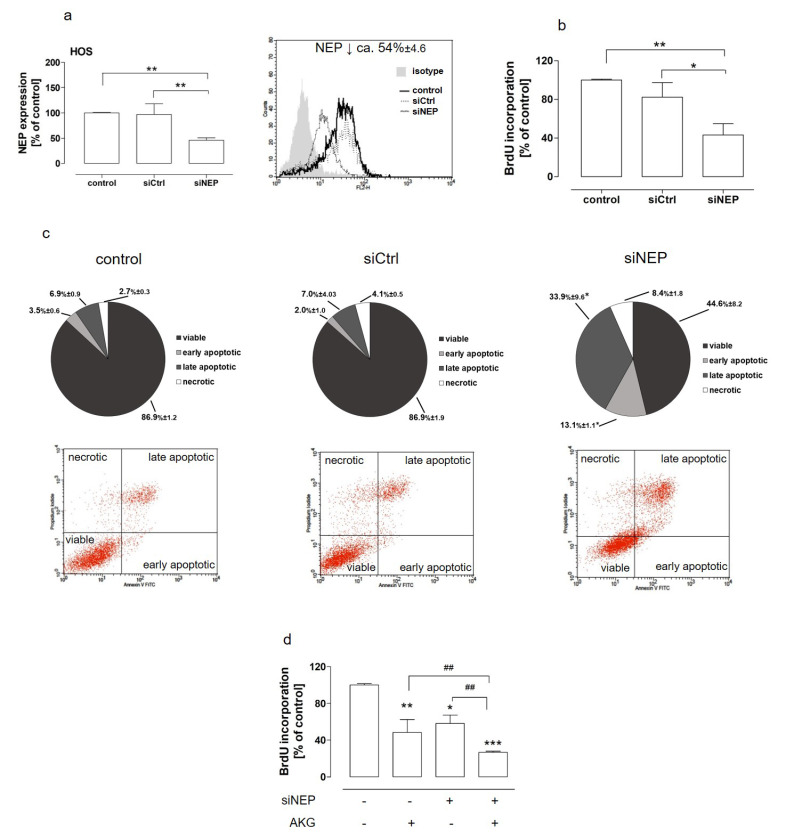
NEP was implicated in growth and in the AKG-mediated antiproliferative effects towards osteosarcoma cells. The level of NEP knockdown in the HOS cells was determined by flow cytometry (**a**). The proliferation and apoptosis of the NEP-expressing (control and siCtrl) and NEP-silenced (siNEP) HOS cells were examined by means of BrdU (**b**,**d**) and flow cytometry assays after AnnexinV-FITC/propidium iodide staining (**c**), respectively. The cells were treated with 50 mM AKG. Isotype—control, NEP-positive cells stained with PE-conjugated mouse IgG1 isotype control, control—siRNA-untreated NEP-positive cells, siCtrl—negative siRNA-treated cells, siNEP—NEP-silenced cells. The results were expressed as a percent of the BrdU incorporation level in the control cells or as a percent of viable, early apoptotic, late apoptotic, or necrotic cells among all the analyzed cells. The dot plots are representative and correspond with the results presented in the graphs. Statistically significant differences: * *p* < 0.05, ** *p* < 0.01, *** *p* < 0.001 in comparison with the control cells or ^##^ *p* < 0.01 in comparison with the siNEP- or AKG-treated cells (one-way ANOVA followed by Tukey’s post-hoc test).

It was previously shown that the NEP inhibition by specific inhibitors resulted in a decrease in osteosarcoma cell growth, which indicated neutral endopeptidase as a promising target of anticancer therapies in this case [23]. However, taking into consideration the two distinct functions of NEP, which are independent of each other, i.e., an enzyme processing bioactive proteins and a receptor involved in cell signaling, it is reasonable to establish whether the downregulation of NEP expression produced the same outcomes as the inhibition of its enzymatic activity in osteosarcoma cells. Hence, the proliferation and apoptosis induction were examined in the NEP-silenced HOS cells. Neutral endopeptidase silencing was performed by means of RNA interference and checked each time before the assay (Figure 5a). The results presented in Figure 5b showed thatthe NEP depletion resulted in inhibition of the proliferation of the osteosarcoma cells (by approx. 45%). This was accompanied by apoptosis induction, as the total number of apoptotic cells was increased by approx. 25% in the NEP-knockdown HOS cells in comparison with the control NEP-expressing cells (Figure 5c). The further studies were performed to determine whether the NEP downregulation was implicated in the anti-proliferative activity of AKG towards the osteosarcoma cells.To this end, the proliferation of the NEP-expressed and NEP-silenced cells after the AKG treatment was examined. The treatment of the NEP-knockdown osteosarcoma cells with AKG decreased cell proliferation in comparison with the NEP-silenced and AKG-treated cells is presented in Figure 5d.

### 2.4. AKG and the NEP Inhibitor Thiorphan Acted Synergistically towards Osteosarcoma Cells

**Figure 6:**
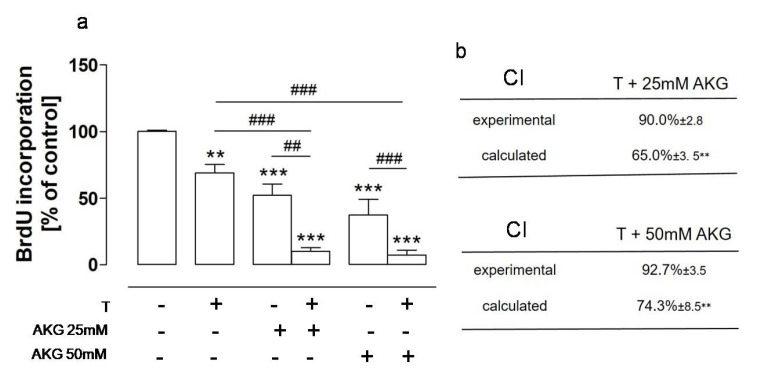
Enhanced antiproliferative activity of combined treatment of osteosarcoma cells with AKG and thiorphan. The HOS cells were treated with thiorphan (T, 500 µM) and AKG (25 mM or 50 mM) for 72 h. The proliferation of the cells was examined by means of the BrdU assay (**a**), and the combination index (CI) was calculated with the method described by Peters et al. [37] (**b**). The results were expressed as a percent of the BrdU incorporation level in the control cells. Statistically significant differences: ** *p* < 0.01, *** *p* < 0.001, in comparison with the control cells or in comparison with the experimental combination index; ^##^ *p* < 0.01, ^###^ *p* < 0.001 in comparison with T- or AKG-treated cells, respectively (one-way ANOVA followed by Tukey’s post-hoc test).

The gold standard in cancer treatments is the use of a combination of two or more therapeutic methods, which has many advantages, e.g., higher effectiveness in comparison with monotherapies [31,32]. Another therapeutic approach in cancer treatment is drug repositioning, which involves the repurposing of drugs approved for treatment of non-cancerous diseases to cancer therapies [33,34]. Moreover, there have been many attempts to exploit natural products, e.g., chemosensitizing agents, in combination therapies of cancers [35]. As a natural substance with suggested anticancer activities acting on diverse targets in cancer cells, AKG is a promising agent to be used in combination with other drugs in anticancer therapies. Moreover, the results presented in this paper, and previously published findings [23], indicated that NEP had a tumor-promoting role in osteosarcoma and that thiorphan, i.e., an inhibitor of NEP enzymatic activity used as an active component of approved antidiarrheal drugs [23,36], exerted moderate antiproliferative activity towards HOS cells. Therefore, further studies were aimed at examining whether AKG in combination with thiorphan produced better outcomes against osteosarcoma cells then when used alone. To this end, the HOS cells were treated with AKG at concentrations that downregulated the level of NEP, i.e., 25 mM and 50 mM, and thiorphan at the highest non-cytotoxic antiproliferative concentration, i.e., 500 µM. The results indicated an enhanced antiproliferative effect of both agents against osteosarcoma HOS cells. A statistically significant decrease in BrdU incorporation was observed in the AKG- and thiorphan-treated cells in comparison with cells treated with a single agent (Figure 6a). Moreover, the calculations based on the method described by Peters et al. [37] revealed that the combined treatment with thiorphan and AKG had a synergistic effect, as the values of the combination index (CI) calculated for all AKG and thiorphan combinations were significantly lower than that obtained experimentally (Figure 6b).

## 3. Discussion

It is known that natural products and their derivatives are extensively investigatedin relation to whether they can be used as effective anticancer drugs, or at least agents supporting the activity of approved anticancer therapies, e.g., as chemo-sensitizing agents. The latter function is achieved among others by reversing or alleviating the disadvantageous effects of standard anticancer therapies, i.e.,the toxicity of conventional cytostatics or chemoresistance and immunotolerance of cancer cells [31,32,33,35,38]. The main features of natural products predisposing them to be used in medicine include high biodiversity, good oral bioavailability, and relatively low intrinsic toxicity. They constitute diverse groups of unaltered or chemically modified natural products (semi-synthetic products), defined mixtures of botanical drugs, or totally synthetic compounds based on natural pharmacophores. It should be noted that over 70 of the currently approved and used chemotherapeutic agents are derived from natural sources, e.g.,taxol, vincristine, irinotecan, etoposide, and paclitaxel. Although most of the natural drugs and supportive agents are derived from plants, other sources, e.g., microorganisms and animals, are also rich in such substances [2].

Epigenetic changes, which are prerequisites for many malignancies, are potentially reversible, in contrast to genetic mutations, and they can be restored to the normal state. Hence, it is reasonable to search for therapeutic compounds with activities influencing epigenetic modifications. Natural substances are widely explored asepigenetic modulators of gene expression; however, as a natural intracellular co-substrate for 2-oxoglutarate-dependent dioxygenases (2-OGDDs), alpha-ketoglutarate (AKG) seems to be especially interesting in this case [3,4,39]. It regulates the activity of the Jumonji C domain containing lysine demethylases (KDM2-7) and ten–eleven translocation hydroxylases (TET1-3), i.e., enzymes involved in histone and DNA demethylation, respectively [3,4,40]. Another mechanism by which AKG influences the epigenetic regulation of gene expression is its indirect impact via hypoxia-inducible factor-1 (HIF-1) on the activity of histone deacetylases (HDAC). Namely, AKG activating prolyl hydroxylases PHD1-3 promotes the degradation of cancerogenic factor HIF-1, which in turn prevents direct HIF interaction with HDAC and allows histone deacetylase to bind to gene promoters [41,42]. However, these are not the only activities of AKG, as it has recently been shown to be a pleiotropic, health-supporting, and therapeutic molecule with low toxicity and good bioavailability; hence, it can be used in medicine [4,43,44].

Taking into account the above-mentioned activities of AKG and the fact that the expression of neutral endopeptidase implicated in the growth and metastasis of cancer cells is regulated epigenetically, we explored the potential effect of AKG on the expression of NEP in cells derived from cervical cancer, colon cancer, and osteosarcoma as well as normal epithelial cells of the intestine and osteoblasts. This study is the first to report that AKG had an impact on NEP expression at the protein level. To date, most studies have been focused on the upregulation of NEP as an anticancer target [13]. To the best of our knowledge, our results showed for the first time a naturally occurring molecule with the ability to downregulate the NEP level in cancer cells, without any significant influence on normal cells. It should be underlined that AKG had an influence on NEP in only one of the tested osteosarcoma-derived cell lines, namely the HOS cell line, and did not change the level of NEP in the cervical and colon cancer cell lines. These preliminary results suggested that the effect of AKG on the NEP expression may have been dependent on whether cells have undergone neoplastic transformation (HOS vs. hFOB), the type of cancer (osteosarcoma vs. cervical and colon cancer), and finally the phenotype and/or stage of cancer (HOS vs. Saos-2). On the other hand, the influence of AKG seemed to be independent of the intrinsic level of neprilysin in cancer cells, because neither up- nor downregulation was observed in cancer cells not expressing NEP (CaSki), expressing a low level of NEP (SiHA and SW948), or expressing a high level of NEP comparable to that observed in HOS, respectively. These preliminary results seemed to be in line with the finding that the NEP expression is regulated epigenetically, i.e., through mechanisms that differ substantially in the case of cancer cells and their normal counterparts or in cells of the same type of cancer exhibiting different phenotypes [30,45,46]. However, this issue needs further investigation.

There are several mechanisms of the epigenetic control of gene expression, e.g., DNA and histone methylation. The effect of epigenetic modifications of histones depends on several aspects: the site, degree, and symmetry of methylation. Histone mono-, di-, or trimethylation may occur on either arginine or lysine residues, which result in specific outcomes, e.g., the methylation of lysine 4 in histone H3 (H3K4) is related to gene activation, while the methylation of lysine 27 in histone H3 (H3K27) is related to gene silencing. In the case of NEP, such modifications along with the methylation of CpG islands in the NEP promoter region are implicated in its expression [13,17,18,26]. To gain insight into the potential epigenetic targets of AKG in the control of NEP expression, our subsequent studies were aimed at the examination of the trimethylation status of histone H3 at two different positions, i.e., lysine 4 (activating) and 27 (suppressing). Taking into account the outcomes of hypermethylation at H3K4 and H3K27 and the indirect influence of endogenous AKG on the activation of lysine demethylases (KDM2-7), the present results might seem to be surprising because the treatment of the HOS cells with AKG did not change the trimethylation level at the H3K4 position, in contrast to the trimethylation status at the H3K27 position, which increased. However, a study conducted by Wang et al. reported that, similar to the H3K27me3 modification considered as silencing expression, the levels of methylation at position H3K9me2 significantly increased. In turn, the methylation level at H3K4me3 (modification considered as activating) was diminished in mouse primary cortical and hippocampal neurons after hypoxic treatment, which was concomitant with a decreased level of NEP [47]. Moreover, the form of AKG used should be taken into account while discussing results obtained in various studies. Namely, in this study, AKG was used as a base form derived from disodium salt. In turn, esterified forms of AKG, e.g., dimethyl-α-ketoglutarate and octyl-α-ketoglutarate, were used in other studies and reported a decrease in H3 methylation at lysine positions after treatment with AKG derivatives. According to a recent study conducted by Parker et al., the form of AKG determined the influence of treatment with exogenous AKG on cellular processes and final outcomes in cells. It was indicated that AKG derived from the base form influenced cytosolic/nuclear dioxygenase activity, whereas AKG derived from esters influenced mitochondrial TCA cycle metabolism [48]. Hence, this issue needs further examination.

This unexpected result prompted us to conduct further studies focused on changes in the methylation pattern of histones after AKG treatment induced by methyltransferases, which implicated them in cancerogenesis via gene silencing. An enhancer of zeste homolog 2 (EZH2), i.e., a methyltransferase as a catalytic subunit of polycomb repressive complexes 2 (PRC2), catalyzed the trimethylation of histone H3 at lysine 27. Hence, the involvement of this methyltransferase was examined to elucidate the possible mechanism of the AKG-induced increase in H3K27me3. The results suggested that EZH2 was partially implicated in the AKG-mediated hypermethylation of H3K27 but not in the AKG-induced NEP downregulation in the HOS cells. As no evidence suggesting the direct involvement of AKG in the EZH2 function has been available to date, this study is the first to indicate that AKG exerted an influence on this methyltransferase [49]. However, this issue needs further study.

It is known that AKG exerts antiproliferative activities towards cancers cells, including osteosarcoma [4,50] and, as presented above, downregulates the expression of neprilysin in HOS cells. Previously published data indicated that the anti-cancer effects on osteosarcoma based on NEP regulation can be achieved via inhibition of its enzymatic activity by specific inhibitors, e.g., thiorphan [23]. However, to date, it has not been indicated whether the same effect can be achieved via NEP downregulation. Hence, to elucidate this issue, proliferation and apoptosis in NEP-silenced HOS cells were investigated. The results indicated that the downregulation of NEP inhibited the proliferation and induced apoptosis in the HOS cells and was involved in the AKG-induced growth inhibition in the osteosarcoma cells. These results suggest the possibility of using AKG as a modifier of NEP expression for therapeutic purposes.

Various therapeutic strategies are used for treatment of cancers. In many cases, medicines and therapeutic methods have to be combined to achieve the expected therapeutic results. Combination therapies allow evading detrimental effects caused by cytostatics or chemoresistance and immunotolerance of cancer cells. Another promising approach for improving cancer treatment is drug repositioning/repurposing, which is focused on switching the approved pharmaceutical agents from their primary application in non-cancerous diseases to their use in cancer treatment. The main advantages of these agents in searching for new anticancer drugs are their defined safety protocols, pharmacokinetic profiles, and molecular targets [16,31,34,35,38]. The aforementioned trends prompted us to examine whether the combination of AKG and the known non-cancerous drug, thiorphan, could lead to mutual enhancement of their anticancer activity. Although combination therapies consider a use of drugs targeted at different pathways but AKG and thiorphan act on the same molecule, i.e., NEP, two aspects seem to justify this combination. First, NEP can be influenced via two different mechanisms involving its enzymatic activity and receptor function. Secondly, AKG is a pleiotropic agent acting on different cellular targets. Moreover, AKG has been recently proved to have another advantageous feature, namely activities supporting normal cells, including osteoblasts, which is beneficial in anticancer therapies [51]. The preliminary studies presented in this paper indicated for the first time that the combination of AKG and the NEP inhibitor thiorphan acted synergistically towards osteosarcoma cells and exhibited anticancer potential.

## 4. Materials and Methods

### 4.1. Cell Cultures

Human colon cancer cell lines: SW 948 (ATCC, no. CCL-237) and SW620 (ATCC, no. CCL-227), human osteosarcoma cell lines: Saos-2 (ATCC, HTB-85) and HOS (ATCC, CRL-1543), human cervical cancer cell lines: SiHa (ATCC, HTB-35) and CaSki (ATCC, CRM-CRL-1550), human normal cells: epithelial cells of the large intestine (CoTr): CCD 841 CoTr (ATCC, CRL-1807), and osteoblasts (hFOB): hFOB 1.19 (ATCC, CRL-11372) were used in these studies. Both normal cell lines were transformed with thermo-labile virus SV-40. All cell lines, purchased from Sigma-Aldrich (St. Louis, MO, USA), were maintained in appropriate media recommended by ATCC, namely, colon cancer cell lines: L-15 medium; Saos-2 cell line: McCoy’s 5A Modified Medium; HOS and SiHa cell lines: MEM Medium; CaSki cell line: RPMI medium; hFOB cell line: 1:1 mixture of DMEM without phenol red and Ham’s F12 medium, and CoTr: DMEM Medium. All media were supplemented with fetal bovine serum (FBS), 100 U/mL penicillin, and 100 μg/mL streptomycin (Sigma Aldrich), except the medium for hFOB, which contained 0.3 mg/mL of G418. The cell cultures were cultivated in standard conditions at 37 °C, 95% humidity, and with 5% CO_2_, except the SW 620 cell line cultivated without CO_2,_ and hFOB and CoTr cultivated at a permissive temperature of 34 °C.

### 4.2. Reagents

Alpha-ketoglutarate (AKG) was used as a disodium salt dihydrate (Na2AKG × 2H2O; (Sigma-Aldrich). The stock solution (1M) of this agent was prepared freshly before each experiment as described by Kaławaj et al. [50].

DL-thiorphan (T) (Sigma-Aldrich), i.e., a specific inhibitor of NEP, was maintained as a stock solution (100 mM), which was prepared as previously described by Mizerska-Kowalska et al. [22].

The working solutions of the reagents used in these studies were prepared freshly in culture medium supplemented with 2% or 10% FBS, as indicated.

### 4.3. Cytotoxicity Assay

The cytotoxicity assay was conducted to estimate the non-cytotoxic concentrations of AKG against SiHa, CaSki, SW948, SW620, and CCD 841 CoTr cells. Briefly, cells plated in 96-well plates at a density of 3 × 10^5^ cells/mL (SiHa, Caski, SW948), 5 × 10^5^ cells/mL (SW620), and 2.0 × 10^5^ cells/mL (CoTr) were treated with AKG at concentrations of 6.25, 12.5, 25, 50, 75, and 100 mM for 48 h. Untreated control cells (0 mM) were cultivated with appropriate cell culture medium with 2% of FBS. The cytotoxicity of AKG was estimated by means of the LDH assay (Sigma-Aldrich) [24]. The assay was performed according to the manufacturer’s instruction. The optical density was determined with the use of an EL800 Universal Plate Reader (Bio-Tek Instruments, Inc., USA). The level of LDH release was expressed as a percent of untreated cells. The highest concentrations of AKG that were non-cytotoxic to all the cells, i.e., 12.5, 25, and 50 mM, were chosen for further experiments (Supplementary Figure S1).

### 4.4. Flow Cytometry Analysis of NEP Level and Histone H3 Methylation

The detection of NEP by means of flow cytometry was carried out for the quantitative analysis of the NEP protein level upon the AKG and/or EZH2 inhibitor treatments and to control the silencing of NEP expression [24,52,53]. To conduct the quantitative analysis, the sub-confluent cell cultures were treated with AKG (12.5 mM, 25 mM, 50 mM) and/or EZH2 inhibitor EPZ005687 (10 µM) for 72 h in standard conditions. Untreated cells (0 mM) constituted the control. For the NEP level analysis, the control and treated cells were incubated with PE-conjugated mouse anti-human NEP mAb IgG1 (BD Biosciences, Pharmingen™, San Diego, CA, USA), and the control cells were also stained with a PE-conjugated mouse IgG1 isotype control (eBioscience^TM^), according to the manufacturer’s instruction. For the histone H3 methylation analysis, the control and treated cells were fixed with 4.8% paraformaldehyde (Sigma Aldrich) for 15 min at room temperature. After thorough washing with PBS, the cells were permeabilized with 90% ethanol for 15 min on ice. After washing with PBS, the cells were suspended in buffer (0.5% BSA in PBS) containing Alexa Fluor 447-conjugated antibodies anti-H3K4 me3 (0.1 µg/mL) or anti-H3K27 me3 (0.2 µg/mL). The control cells were also stained with Alexa Fluor 447-conjugated IgG1 isotype control used at equivalent concentrations as described above. All antibodies were purchased from Cell Signaling Technology. After 1-h staining at room temperature, the cells were washed with antibody dilution buffer. All data were acquired on a FACS Calibur (BD Biosciences) and analyzed using Cell Quest soft-ware Pro Version 6.0. for the Macintosh operating system (BD Biosciences). The cells were considered as NEP- or respective modification of histone-positive if the MFI level in cells labeled with NEP or appropriate histone modification-specific antibodies was significantly higher than the MFI level in cells labeled with the isotype control. The levels of NEP or H3K4/27 me3 were expressed as a percent of control cells and/or as relative mean fluorescence intensity (MFI).

### 4.5. NEP Gene Expression Silencing

The knockdown of NEP gene expression was achieved by siRNA (Silencer^®^ Select Invitrogen^TM^ThermoScientific, Waltham, MA, USA) against human NEP. For this purpose, HOS cells were plated in 6-well plates at the density of 4.0 × 10^4^ cells/mL and the procedure was conducted as described previously [5]. The effectiveness of NEP silencing was controlled each time by flow cytometry analysis. The average level of NEP silencing was 54%.

### 4.6. Cell Proliferation Assay

The proliferation of HOS was established by means of the BrdU incorporation assay (Roche Molecular Biochemicals, Mannheim, Germany) [22]. To this end, the HOS cells were seeded at the density of 2.0 × 10^4^ cells/mL in 96-well plates. In some experiments, the HOS cells were treated separately or together with thiorphan (500 µM) and AKG (25 mM or 50 mM). In this case, the untreated cells constituted the control. In another study, HOS cells expressing NEP (control and siCtrl) and those after NEP silencing were treated with AKG at concentrations of 50 mM. In all assays, the cells were cultivated in medium with 10% FBS and exposed to all the tested agents for 48 h. Cell proliferation was evaluated according to the manufacturer’s instructions. The proliferative activity of the HOS cells was expressed as a percent of the BrdU incorporation level in the control cells.

### 4.7. Neutral Endopeptidase Activity Assay

The NEP activity in cancer cells after the treatment with AKG was determined using the Neprilysin Activity Assay Kit (BioVision Incorporated, Milpitas, CA, USA) according to the manufacturer’s instructions with modifications [23]. The assay is based on the ability of active NEP to cleave a synthetic substrate (Abz-based peptide) to release a fluorophore whose level is proportional to NEP activity. Briefly, the cell lysates (10 µg of total protein), the control (10ng of NEP), and the background sample (10 µL of assay buffer) were incubated with a NEP substrate solution at the final sample volume of 100 µL at 37 °C for 2 h. Fluorescence was measured at Ex/Em = 330/430 nm, respectively. The assays were carried out at least in triplicates and the results were expressed as a percent of NEP activity in the control (0 mM) cells.

### 4.8. Apoptosis Assays

The NEP-expressing (control and siCtrl) and NEP-silenced (siNEP) HOS cells (1.0 × 10^5^ cells/mL) were cultivated in 6-well plates in culture medium with 2% FBS for 72 h in standard conditions. The quantitative analysis of the cell apoptosis rate was performed using an Annexin V-fluorescein isothiocyanate (FITC) apoptosis detection kit (BD Biosciences, BD Pharmingen™, USA) according to the manufacturer’s instruction [5]. The data were acquired using FACS Calibur and analyzed using Cell Quest Pro Version 6.0. for the Macintosh operating system. The results were expressed as a percent of viable, apoptotic (early + late apoptotic), or necrotic cells among all analyzed cells.

### 4.9. Evaluation of Combination Effects

The effectiveness of the combination treatment of HOS with AKG and thiorphan was evaluated with the method described by Peters et al. [37]. This method assumes that the combined treatment is defined as synergistic if the calculated combination index (CI) is significantly lower than the value obtained experimentally. On the other hand, if the calculated CI is significantly higher, the combined treatment is considered as antagonistic. The CI was calculated using the following formula:

CI = 100 − [(100 − %T) × (100 − %AKG)]/100 (1)

where %T and %AKG correspond with the percentage of proliferation inhibition obtained experimentally for thiorphan (T) and AKG used alone.

## 5. Conclusions

These preliminary studies have shown for the first time that downregulation of the NEP protein level is a promising target in therapies of osteosarcoma. Moreover, this therapeutic goal can be achieved via AKG-induced downregulation of NEP and the synergistic activity of AKG with thiorphan, i.e., a NEP specific inhibitor. Additionally, this study has reported for the first time that exogenous AKG can influence the activity of histone methyltransferase EZH2. However, this issue needs further investigation to elucidate the mechanisms of this phenomenon.

## Data Availability

Not applicable.

## Supplementary Materials

The following supporting information can be downloaded at: https://www.mdpi.com/article/10.3390/molecules28010097/s1, Figure S1: Cytotoxic effect of AKG on cervical cancer cells SiHa and CaSki, colon cancer cells, SW948 and SW620, and normal large intestine epithelial cells CoTr.
